# Proteolytic *Bacillus* sp. Isolation and Identification from Tannery Alkaline Baths

**DOI:** 10.3390/molecules30173632

**Published:** 2025-09-05

**Authors:** Manuela Lageiro, Fernanda Simões, Nuno Alvarenga, Alberto Reis

**Affiliations:** 1 National Institute of Agricultural and Veterinary Research (INIAV), Unit of Technology and Innovation, 2780-157 Oeiras, Portugal; fernanda.simoes@iniav.pt; 2 NOVA School of Science and Technology (NOVA FCT), NOVA University Lisbon, 2829-516 Caparica, Portugal; 3 GeoBioTec Research Center, NOVA University Lisbon, 2829-516 Caparica, Portugal; 4 Laboratório Nacional de Energia e Geologia IP (LNEG), Unidade de Bioenergia e Biorrefinarias (UBB), 1649-038 Lisboa, Portugal; aberto.reis@lneg.pt

**Keywords:** proteases, *Bacillus subtilis*, enzymatic activity, stain removal, leather, detergent, bio-economy

## Abstract

The application of microbial alkaline proteases holds significant potential for eco-sustainable industrial processes by reducing chemical usage and lowering the costs of effluent treatment. In the search for novel proteases with industrial relevance, several microbial strains were isolated from alkaline baths of the Portuguese tannery agroindustry. The most promising protease-producing strains were selected for identification and further study. Two isolates demonstrated the highest proteolytic activity, reaching 0.51 ± 0.01 U mL^−1^ and 0.70 ± 0.01 U mL^−1^ after 7.5 h of submerged cultivation in nutrient broth. Based on API biochemical tests, molecular biology techniques, and GC-FAME analysis of membrane lipids, the isolates were identified as *Bacillus subtilis* and incorporated into INIAV’s collection of industrial microbial cultures as *B. subtilis* CCMI 1253 (BMR2) and *B. subtilis* CCMI 1254 (BMR1). The most promising protease producer, *B. subtilis* CCMI 1253 (BMR2), exhibited a maximum specific growth rate of 0.88 ± 0.10 h^−1^. The proteases produced exhibited good extracellular proteolytic activity, with adaptability to industrial conditions, indicating their suitability for agroindustry applications such as leather making, detergent formulations and the treatment of effluents and protein residues. The results support the potential of microbial proteases as valuable tools in the bioeconomy and green chemistry.

## 1. Introduction

Proteolytic microorganisms produce proteases, a class of enzymes that degrade proteins. Proteases are one of the three most important groups of industrial enzymes, accounting for approximately 60% of the world’s enzyme market in terms of sales [1,2,3]. Among industrial proteases, 35% are alkaline proteases [4].

Microorganisms are a cheaper source for enzyme production due to their rapid growth, the limited space required for culture, and their potential for genetic manipulation to produce new enzymes with high stability and specific activities [3,5]. In addition, nutritional and cultural parameters such as the growing media formulation (carbon, nitrogen, and ion sources), pH, temperature, and incubation time also play an essential role in protease production [3,6].

As proteolytic microorganisms, the *Bacillus* genus comprises aerobic or facultatively anaerobic, catalase-positive, rod-shaped, spore-forming bacteria with a wide diversity, consisting of phenotypically and genotypically heterogeneous species [7,8,9]. These bacteria are found in diverse environments, particularly in soil [10], but also in extreme environments with high alkalinity, temperature, and/or salinity [11].

Fermentation involves multiple reactions that convert complex substrates into simpler compounds, encompassing microbiological, enzymatic, chemical, biochemical, and physical processes. These complex biosystems contain enzymes from both raw materials and microorganisms that are responsible for hydrolysis reactions, such as proteolysis [12,13,14]. Some microbes produce extracellular proteases, which are either bound to the microbial cell envelope or secreted to the medium. These proteases degrade environmental proteins to facilitate microbial growth [15].

The production of extracellular proteases is associated with the end of the exponential phase, as their expression is regulated by nutrient availability, and they are produced to degrade insoluble proteins present around cells in natural habitats [16].

Protease applications are vast, and several applications have been described in the literature, which can be categorised into nonfood and food applications [2]. Nonfood applications concern detergents [17,18], leather making [19,20,21,22,23], silk degumming [24,25,26], wool felting prevention [27], medicines [2], pharmaceuticals [4,28,29,30], silver recovery from X-ray films [28], and proteaginous waste and effluent treatment [31]. Food applications concern cheese production in the dairy industry [26], meat tenderisation [2,26,32], baking [33,34], and gluten-free products [35].

While several microorganisms are well-known as a source of industrial proteases [36], several works have been conducted on systematically screening proteolytic isolates from tannery effluents or waste environments [37,38,39,40,41,42,43,44], as well as leather by-products [45]. However, to our knowledge, no work has been conducted on screening isolates from tannery soaking, liming and purge baths, which are unique due to their exposure to protein-rich waste, harsh chemicals and environmental conditions. In these industrial environments, strains with enhanced protease production and tolerance to industrial processing conditions can exist, and their screening is valuable for applications in leather processing, detergent industry, effluent treatment and waste management.

Lageiro et al., 2024 and 2025 [46,47], previously studied protease applications using the bulk proteolytic broth produced in a bioreactor by the isolated microorganism with higher proteolytic activity, analysing the proteolytic broth application in the leather tanning process and the removal of stains from cotton fabric [46,47].

The present work addresses the screening and isolation of microbial proteolytic strains from alkaline baths of the Portuguese tannery agroindustry, selected for their high protease activity and rapid growth in specific proteolytic indicator media such as skim milk casein, followed by the identification of the most proteolytic isolates based on their physiological, biochemical, and genetic characteristics, using API (Analytical Profile Index) tests, GC-FAME (Gas Chromatography of Fatty Acid Methyl Esters) analysis of cell membrane lipids, and 16S rDNA gene sequencing. The selected isolates with high proteolytic potential were affiliated with the *Bacillus subtilis* taxonomic group and deposited at the INIAV Culture Collection of Industrial Microorganisms. The isolates’ proteolytic activity and cell growth were characterised. The *Bacillus* genus includes numerous species that are often defined as excellent sources of extracellular proteases [11] and have been widely studied, holding significant commercial, industrial, and biomedical importance [2].

## 2. Results

The screening of microorganisms from industrial tanning baths of Portuguese agroindustry was performed to obtain strains with proteolytic activity already adapted to industrial alkaline conditions.

### 2.1. Screening of Proteolytic Microorganisms from Tanning Baths

The microorganism screening was performed using soaking, purge, and liming alkaline baths from a Portuguese tanning agroindustry with pH levels of 9.03, 9.45, and 12.62, respectively. The screening results are presented in Table 1.

The NA medium inoculated with the soaking and the purge baths showed high growth of microorganisms in the Petri dishes.

The alkaline *Bacillus* medium (ATCC 661) only showed growth with the liming bath inoculum due to the bath’s high alkalinity.

For the SMA medium inoculated with the soaking bath, the growth was uncountable (CFU > 300) for the dilution (10^−2^) used in Table 1 results (the subsequent dilutions were countable). However, it presented seven colonies with halo formation (clear areas around the colonies due to milk casein degradation). The halos obtained for the soaking bath isolates were smaller than the ones obtained for the purge bath isolates. The SMA medium inoculated with the purge bath yielded eleven colonies. Four of them presented the biggest halo formation after 24 h of inoculation. Those four proteolytic colonies (four CFU with the larger halos) from the purge bath (pH 9.45) were used to isolate four microorganisms with caseinolytic properties (observed by the formation of halos in SMA solid medium), designated BMR1 to BMR4, and selected for further characterisation of their proteolytic activity and growth.

The absence of growth in the YM agar medium indicated that yeasts were not isolated from any of the tested alkaline tanning baths (Table 1).

The halo dimensions of the four isolates from the purge tanning bath, which presented the largest proteolytic halos in SMA medium at 37 °C, are shown in Table 2. BMR1 and BMR2 exhibited the larger colonies and the larger proteolytic activity halos (caseinolytic activity) with halo diameters of 16.9 ± 2.2 mm and 30.1 ± 1.4 mm, respectively.

Isolates’ growth in the NA and SMA media was lower at 30 °C than at 37 °C, and no growth occurred at 50 °C. The microorganisms’ growth was faster in the NA medium than in the SMA medium.

Four microorganisms with proteolytic activity were isolated from the purge tanning bath, and those with the highest proteolytic activity (BMR1 and BMR2) were further identified as *Bacillus subtilis*, a GRAS (Generally Recognised as Safe) [48] microorganism.

### 2.2. Proteolytic Activity and Growth of the Isolated Microorganisms in Solid and Liquid Medium

**Figure 1:**
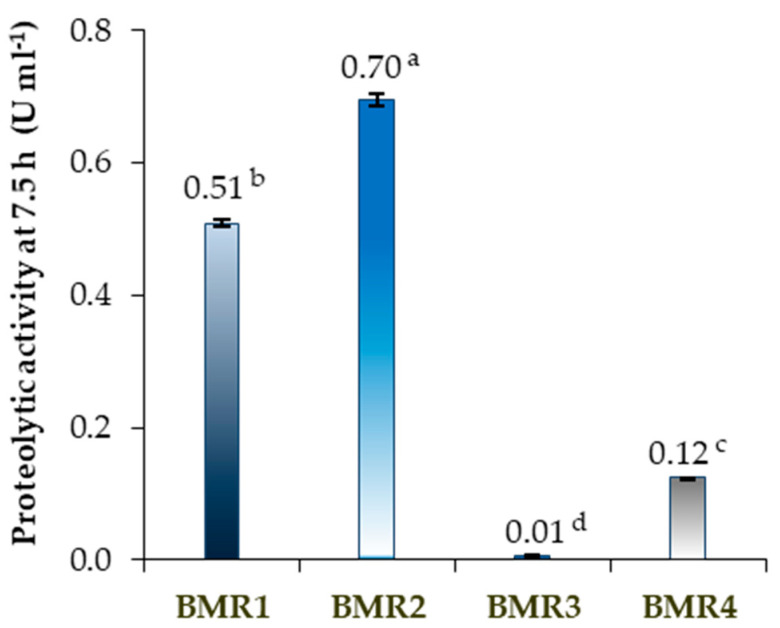
Proteolytic activity (U mL^−1^) of the isolated proteolytic microorganisms BMR1 to BMR4 from the purge tanning bath at 7.5 h fermentation time. Different letter indexes indicate significant differences near the average mean, as determined by ANOVA and the Tukey HSD test (*p* < 0.05, *n* = 3), with error bars representing standard deviations. One unit (U) produced by the hydrolysis of casein at a wavelength of 660 nm is equivalent to 1.0 µmol of L-tyrosine per minute at pH 7.5 and a temperature of 37 °C [49,50].

The achieved proteolytic activities at 7.5 h fermentation time are presented in Figure 1. The purge bath isolates BMR1, BMR2, and BMR4, which exhibited the maximum proteolytic activity for 7.5 h of incubation time. However, isolate BMR3 only showed a maximum proteolytic activity of 0.11 ± 0.00 U mL^−1^ at 15 h fermentation time, an activity similar to that presented by isolate BMR4 at half the time (Tukey HSD test, *p* < 0.05, *n* = 3).

At 7.5 h, the isolated BMR1 and BMR2 achieved the highest proteolytic activity (0.51 ± 0.01 U mL^−1^ and 0.70 ± 0.01 U mL^−1^, respectively). The isolate’s proteolytic activities in the liquid nutrient medium (submerged fermentation) correlated with the observed dimensions of the halos formed on the SMA plates.

**Figure 2:**
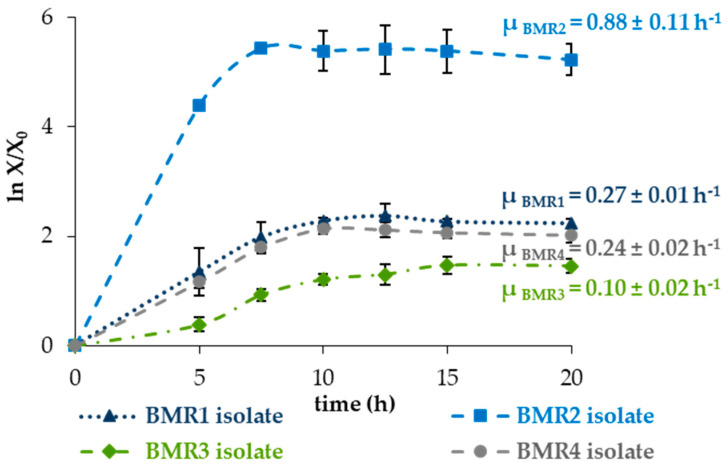
Growth assessment of BMR1 and BMR2 isolates. µ: maximum specific growth rate. X: cell concentration in cell number counting for each time. X_0_: cell number counting after inoculation. The points represent the average data from two parallel growth experiments for each isolate, with standard deviation error bars.

The specific growth rates (μ) of BMR1 to BMR4 isolates were determined in liquid nutrient medium as described in the methods, and their growth curves are shown in Figure 2.

The submerged culture of the BMR2 isolate showed the most significant maximum specific growth rate of 0.88 ± 0.11 h^−1^, followed by the BMR1, BMR4 and BMR3 isolates with 0.27 ± 0.01 h^−1^, 0.24 ± 0.02 h^−1^, and 0.10 ± 0.02 h^−1^, respectively. The end of the exponential phase occurred at approximately 7.5 h for BMR1, BMR2 and BMR4 isolates, coinciding with the time at which they achieved the maximum protease activity. The BMR3 isolate showed a bigger lag phase with a later end of the exponential phase, suggesting that maximum protease production was associated with the end of the exponential phase for all the isolates.

The BMR1 and BMR2 isolates were then selected for microbial identification due to their high extracellular proteolytic activities and growth performance.

### 2.3. Microorganism Identification

The selected microorganisms isolated from the industrial purge bath, BMR1 and BMR2, were subjected to several characterisation tests, including morphological, physiological, biochemical, molecular and GC-FAME lipids analysis.

#### 2.3.1. Morphological and Physiological Characterisation

The selected isolated microorganisms, as determined by Gram and spore detection tests, as well as microscopic assessment for morphological identification, were found to be Gram-positive, large, rod-shaped bacteria with rounded ends, often found in pairs or chains, and capable of forming endospores. The malachite green staining test resulted in bacilli stained red (due to safranin counterstaining) and spores stained green under microscopic visualisation. Spores were found to be ellipsoidal, and when inside the cells, they were located in central or paracentral positions. The results from microscope visualisation, Gram staining, spore formation and colony analysis are summarised in Table 3.

**Figure 3:**
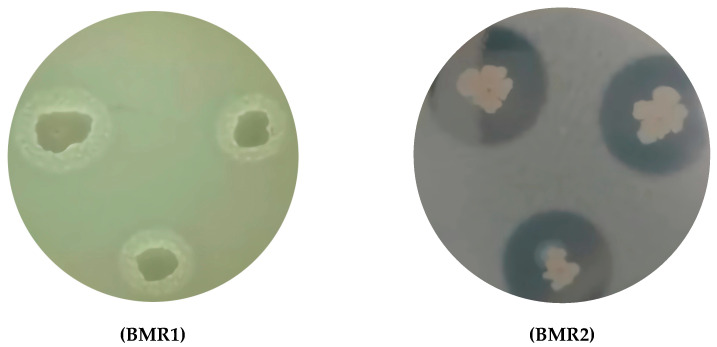
BMR1 and BMR2 on SMA medium after 24 h incubation at 37 °C. Both isolates exhibit irregular colony borders and proteolytic halos. The BMR2 isolate presented the largest halos.

The colonies in SMA medium plates of the BMR1 and BMR2 isolates exhibited irregular mucous colony borders and large proteolytic translucent halos, as shown in Figure 3.

The cells of the BMR1 and BMR2 isolates differed in mobility and visual aspects of the former colonies. The BMR2 isolate cells exhibited greater mobility and formed the largest colonies, accompanied by the largest proteolytic halos due to casein degradation in the SMA medium. Measured halo dimensions are presented in Table 2.

In submerged culture, the BMR2 isolate demonstrated higher proteolytic activity and faster growth than the BMR1 isolate, as shown in Figure 1 for proteolytic activity and Figure 2 for growth assessment.

**Figure 4:**
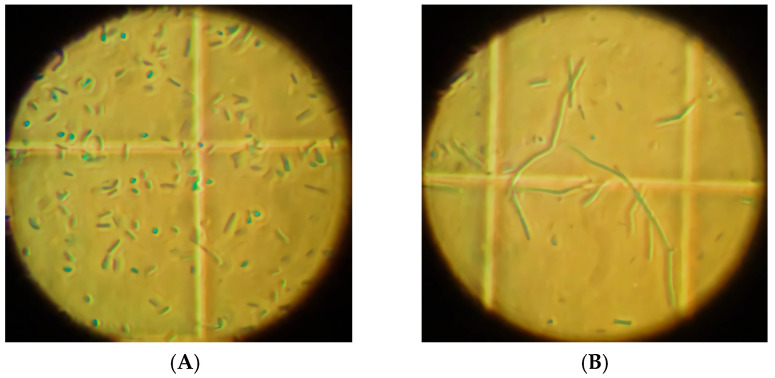
Thoma chamber microscope visualisation showing (**A**) the rod-shaped cells with the spores and (**B**) the cell chains for the BMR2 isolate (Phase-contrast optical microscope with 400× magnification).

The rod-shaped cells with the spores and the cell chains of the higher proteolytic isolate, BMR2, are presented in the pictures of Figure 4.

#### 2.3.2. Biochemical Tests

The API 20E test (BioMérieux) to identify and differentiate members of the Enterobacteriaceae family, and the API 50CHB/E test (BioMérieux) to identify *Bacillus* related genera and Gram-negative rods, allowing fermentation of 49 carbohydrates, were used to obtain the results presented in Table 4; the tests with negative results for both isolates were not listed.

The API 20E and API 50CH test results were entered into the software APILAB Plus v 3.2.2, which revealed that the isolated microorganisms BMR1 and BMR2 showed 98.6% and 99.2% similarity to *Bacillus subtilis*, respectively.

#### 2.3.3. Molecular Identification by 16S rRNA Gene Sequencing

The molecular identification of the selected proteolytic isolates, BMR1 and BMR2, was confirmed through 16S rRNA DNA sequencing. PCR products were visualised after 1.2% agarose gel electrophoresis, showing 1429 and 1423 base pairs for BMR1 and BMR2, respectively.

A BlastN search of the sequenced base pairs region of 16S rRNA in NCBI GenBank revealed more than 99% genome similarity with *Bacillus subtilis* strains (NCBI Blast at https://blast.ncbi.nlm.nih.gov, accessed on 25 March 2025). The sequences were deposited in GenBank with accession numbers PQ326426 and PQ325245 for BMR1 and BMR2, respectively.

The isolates BMR1 and BMR2 showed high similarity (99.23% and 99.72%, respectively) to *Bacillus subtilis* JCM 1465 and *Bacillus subtilis* DSM 10 strains (GenBank Accession numbers NR_113265.1 and NR_027552.1) retrieved from NCBI Blast (https://blast.ncbi.nlm.nih.gov, accessed on 25 March 2025).

BMR1 and BMR2 presented 99.86% identity between them, as indicated in the identity matrix, which was presented in the Supplementary Materials Table S1. The matrix was created with sequences retrieved from NCBI Blast due to their high similarity (nucleotide identity > 98%), using as an outsider a *B. cereus* (Table S1).

**Figure 5:**
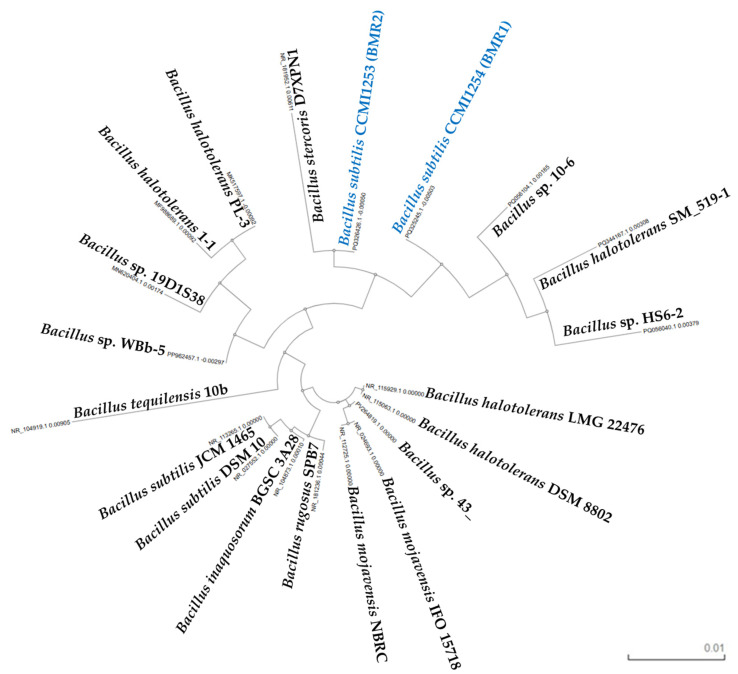
Phylogenetic tree of the *Bacillus subtilis* isolates from Clustal Omega Multiple Sequence Alignment (MSA) [52] (EMBL-EBI, European Molecular Biology Laboratory, Cambridgeshire, UK) with 20 GenBank accession numbers (Table S1) sharing the highest identity percentages (sequences retrieved from NCBI Blast, https://blast.ncbi.nlm.nih.gov, accessed on 25 March 2025).

Multiple sequence alignment of the isolated *Bacillus subtilis* sequences and other *Bacillus* strains sequences (nucleotide identity > 98%, Table S1) was performed based on seeded guide trees and Hidden Markov Models (HMM) profile-profile techniques at Clustal Omega Multiple Sequence Alignment (MSA) [52], and visualised by the phylogenetic tree represented in Figure 5. Both isolates clustered within the *B. subtilis* clade, closely related to *B. subtilis* DSM 10 and ICM 1465.

The phylogenetic tree (Figure 5) illustrates the evolutionary relationships among the isolates and reference *Bacillus* species. The topology of the tree indicates a strong genetic affiliation between the isolates and the *B. subtilis* lineage. These results provide strong evidence that isolates BMR1 and BMR2 are affiliated in the *Bacillus subtilis* taxonomic group.

#### 2.3.4. Fatty Acid Methyl Esters Analysis

To confirm the previous bacterial identification of the isolated BMR2, Gas Chromatography Analysis of the cell Fatty Acid Methyl Esters (GC-FAME) was also performed, resulting in a profile with nine reference peaks presented in Table 5. The GC-FAME profile of the BMR2 isolate was searched in the Sherlock version 4.0 Microbial Identification System (MIDI Inc., Newark, DE, USA), yielding a high similarity index of 0.893 with *Bacillus subtilis*.

The isolate BMR2 had a *C15 iso*/*C15 anteiso* ratio of 0.31, confirming its affiliation with Cluster I (typically characterised by a *C15 iso*/*C15 anteiso* ratio > 0.30), with *B. subtilis* presenting the lowest *C15 iso*/*C15 anteiso* ratio among *Bacillus* species in Cluster I.

Due to the high similarity and nucleotide identity of BMR1 and BMR2 isolates, with high similarity and nucleotide identity to *Bacillus subtilis* strains obtained by all the characterisation tests, they were identified as *Bacillus subtilis* and registered and deposited in the Collection of Industrial Microorganisms Cultures (CCMI) of the Portuguese National Institute for Agricultural and Veterinary Research, INIAV, as *Bacillus subtilis* CCMI1254 (BMR1) and *Bacillus subtilis* CCMI1253 (BMR2), respectively.

## 3. Discussion

### 3.1. Screening of Proteolytic Microorganisms

Proteolytic microorganisms are commonly found in leather industry environments, especially in leather baths, wastes and effluents. These microbes produce proteases, playing a key role in breaking down protein-rich waste and can be both beneficial for effluent and waste management and possibly problematic for leather quality. The screening of proteolytic microorganisms from the leather industry was performed to produce proteases adapted to these industrial, harsh conditions and *four* bacteria were isolated from a purge Portuguese industrial leather alkaline bath presenting caseinolytic activities (Table 1 and Table 2), two of them with high extracellular proteolytic activities of 0.51 U mL^−1^ (BMR1 isolate) and 0.70 U mL^−1^ (BMR2 isolate) in 7.5 h (Figure 1).

Masi et al., 2021 [37] isolated bacteria from leather industrial effluent collected from the Modji leather industry at Modji, Ethiopia and selected three potential alkaline protease strains. The best alkaline protease-producing bacteria showed extracellular protease activity of 19 U mL^−1^ in 48 h, presenting more activity than BMR2 but more than 6 times the fermentation time the BMR2 isolate took to achieve the maximum protease activity. The best protease producer isolated by them [37] was identified as a *Bacillus cereus* strain (with 97.9% similarity) based on morphological and biochemical characteristics, as well as molecular identification using the 16S rRNA gene sequence.

Ashraf et al., 2023 [53] isolated a *Pseudomonas aeruginosa* strain SM4 from tannery waste and demonstrated that it produced robust extracellular proteases at 105 U mL^−1^ within 30 h. These enzymes were effective in degrading protein waste and could be used to enhance leather processing while reducing pollution [19].

*Bacillus* and *Pseudomonas* species are some of the most commonly identified and effective proteolytic microorganisms in leather baths and effluents. They play a crucial role in protein degradation and waste management and can be harnessed for more sustainable leather processing. Other genera also contribute to the diverse proteolytic community in these environments. Additional proteolytic bacteria identified in leather environments, including *Proteus*, *Serratia*, *Klebsiella*, *Providencia*, *Achromobacter*, *Enterobacter*, *Myroides*, *Acinetobacter*, and *Pseudomonas* exhibited extracellular protease production [53,54].

Among the *Bacillus*, the Gram-positive *Bacillus subtilis* species is known as a GRAS (Generally Recognised as Safe) microorganism [48,55,56] by the Food and Drug Administration (FAO) and an important producer of extracellular proteases [57] with several industrial applications in the food industry [2], feed production [58], leather tanning [4,59], detergent production [4], textile production [4], pharmaceutical industry [2,4], health [60], protein hydrolysis [2,15], waste and effluent treatment [31,61,62].

*Bacillus subtilis* is often referred to as a *workhorse* microorganism in biotechnology, mainly because of its natural production capacity and adaptability [9,63]. *Bacillus subtilis* grows rapidly in a short fermentation cycle, typically around 48 h [48]. These bacteria exhibit an excellent protein secretion ability, making them an important host for the production of medicinal proteins and industrial enzymes [64]. Su et al., 2020 [48], presented a schematic diagram of *B. subtilis* protein secretion pathways [48]. The bacteria’s highly efficient protein secretion system and adaptable metabolism have been widely utilised as a cell factory for the microbial production of chemicals, enzymes, and antimicrobial materials for industry, agriculture, and medicine [48].

Tannery wastewater microbiota was screened for metagenome derived protease (PersiProtease1) detergent protease by Ariaeenejad et al., 2022 [65], and the protease was efficiently applied for biodegradation of real sample tannery wastewater protein, chicken feathers, whey protein, dehairing sheepskins, and waste X-ray films.

Enzymes play a crucial role in the leather manufacturing industry, providing significant economic benefits and positive environmental consequences [66].

### 3.2. Proteolytic Activity and Growth

*B. subtilis* were isolated and studied for protease production by several authors from industrial effluents [59], marine sediments [25], and extreme environments such as alkaline or saline soils [11], with maximum proteolytic activities ranging 0.89–2.5 U mL^−1^ after 24 to 72 h of submerged fermentation, depending on the cultivation medium and strain specific characteristics [17,21,57]. *B. subtilis* produces different extracellular enzymes, including six different proteases, an α-amylase, a levansucrase, several β-glucanases and lipolytic enzymes [67]. The protease activity is specific to the microbial strain rather than to the species or the isolated origin of the microbe [15].

In this study, bacteria isolated from the tannery alkaline purge bath produced extracellular proteolytic enzymes with 0.12–0.70 U mL^−1^ proteolytic activity within only 7.5 h of submerged fermentation using nutrient broth. They present promising characteristics as they growth fast and are producers of proteases with good activities, especially the BMR2 isolate. The *Bacillus subtilis* bacteria are known for their rapid grow in a short fermentation cycle of around 48 h [48]. The BMR1 to BMR4 isolates presented a shorter fermentation cycle of fewer than 20 h, with BMR1, BMR2 and BMR4 achieving the end of the exponential phase at 7.5 h (Figure 1 and Figure 2).

Rapid growth is beneficial for shortening the fermentation period and enhancing the production of the target product [68]. The BMR2 isolate specific growth rate, μ = 0.88 h^−1^, corresponding to a doubling time of 47.5 min (calculated by Equation (2), Section 4. Materials and Methods) in NA medium at 37 °C, was very promising. Despite the specific growth rate depending on fermentation conditions like medium, pH, temperature, aeration, and stirring, the BMR2 isolate specific growth rate is similar to the one of the constructed fast-growing *B. subtilis* (μ = 0.75 h^−1^) using genetic manipulation and laboratory evolution by Liu et al., 2020 [68], from the wild strain *B. subtilis* 168 BS168 (μ = 0.49 h^−1^) [68].

On the growth curve, four distinct phases can be identified when plotting the logarithm of viable cell concentration (X) versus time. First, the lag phase, which occurs after inoculation and persists until the cells have acclimated to their new environment; second, the exponential growth phase, when cell growth proceeds at an exponential rate (indicated by a straight line on the semi-log plot), followed by a stationary phase when the growth is approximately zero because the number of the borning cells is equal to the number of the dying cells; and finally the death phase where some cells lose viability or are destroyed by lysis [69]. When essential nutrients are depleted or the toxic products begin to accumulate, a deceleration phase can be considered between the exponential and the stationary phases [70].

The isolates exhibited maximum proteolytic activity at the end of the exponential phase, suggesting that protease production is related to the end of the exponential phase, as discussed by Abe et al., 2009 [16].

### 3.3. Microorganism Identification

The isolated BMR1 and BMR2 were identified based on their morphological and biochemical characteristics according to Bergey’s Manual of Determinative Bacteriology [71].

Concerning the morphological characteristics of the isolates presented in Table 3 results the isolates exhibit attributes of the *Bacillus subtilis* group, in accordance with the observed by Logan and Berkeley in 1984 [51] for a *Bacillus* study showing for *B. subtilis* strains group, cell width between 0 and 8 µm, 22% of the strains presented formation of cells chains, 95% presented high motility, and 100% presented ellipsoidal spores, being in 40% of the strains in central or paracentral position [51]. Results are in accordance with the morphological characteristics of the *Bacillus subtilis* group reported by other authors [72,73,74,75] and with the results presented in Table 3.

The *Bacillus subtilis* group was previously defined by Gibson in 1944 [76] and Gordon et al. in 1973 [77] and comprised *B. subtilis*, *B. pumilus*, and *B. licheniformis*, with later additions of *B. amyloliquefaciens* and *B. megaterium* [51].

Concerning the biochemical test results presented in Table 4, they are in accordance with those observed by Logan and Berkeley in 1984 [51] for the group of *Bacillus subtilis* strains in their study. The oxidase test for *Bacillus subtilis* can yield positive [72] or negative [78] results, depending on the strain [78]. The isolated BMR1 and BMR2 both tested positive for oxidase. The application of classical phenotypic tests for differentiating *Bacillus* species indicated that only some of them had clearly distinguishing features [79,80].

*B. subtilis* strains exhibit greater similarity in gene sequence-based phylogeny (e.g., 16S rRNA) than in fatty acid methyl ester (FAME) profiles, underscoring that genetic markers provide a more accurate measure of relatedness than chemotaxonomic lipid profiling [81,82]. Roberts et al., 1994 [83] claimed that *B. subtilis* and *B. mojavensis* can be distinguished only by combining different identification methods, the differences in whole-cell fatty acid composition, divergence in DNA sequence, and resistance to genetic transformation between taxa.

*Bacillus subtilis* is distinguished from other *Bacillus* species primarily through a combination of genetic, biochemical, and phenotypic characteristics. While some *Bacillus* species share similarities, *B. subtilis* exhibits unique traits in its genome, cell wall composition, and ability to utilise various carbon sources, but a combination of genetic, biochemical, and phenotypic characteristics is required for its identification [84]. Additionally, specific PCR-based methods targeting genes like *pyrA* and *aroE* can reliably differentiate *B. subtilis* from other *Bacillus* species [85].

The genus *Bacillus* has been the most extensively studied concerning the branched-chain fatty acids [86]. *Bacillus subtilis* exhibits a distinct fatty acid profile characterised by a high content of branched-chain fatty acids, such as iso- and anteiso-fatty acids, which are crucial for its classification and identification [87]. *Bacillus subtilis* reference fatty acids are primarily composed of branched-chain fatty acids (Σ *iso*, *anteiso*) at nearly 95% [88], a characteristic of the *Bacillus* genus [89]. The high proportion of iso- and anteiso-branched fatty acids is a distinguishing feature of the microorganism [90]. These branched structures, derived from branched amino acids, play a crucial role in maintaining membrane fluidity, especially in response to environmental changes such as temperature fluctuations and adaptation to environmental stresses [91]. The major fatty acids include: 25–35% iso-pentadecanoic acid (iso-C15:0), 40–50% anteiso-pentadecanoic acid (anteiso-C15:0); 5–10% iso-hexadecanoic acid (iso-C16:0) and palmitic acid, straight-chain (C16:0); 2–5% anteiso-heptadecanoic acid (anteiso-C17:0). The minor fatty acids (found in smaller proportions) are iso-C14:0, myristic acid (C14:0); iso-17:0 (straight-chain heptadecanoic acid).

Kämpfer, 1994 [87], claimed that the *Bacillus* species could be clustered into subgroups at a similarity level of 97.5% [87]. The ratio of iso-C15:0 to anteiso-C15:0 (*C15 iso/C15 anteiso*) is a critical factor in differentiating *B. subtilis* from other *Bacillus* species. The *Bacillus* spp. main group, with *C15 iso/C15 anteiso* < 2, was divided into two subgroups: the Cluster II, of *C15 iso/C15 anteiso* < 0.3, and the Cluster I, of *C15 iso/C15 anteiso* > 0.3, including *B. amyloliquefaciens*, *B. brevis*, *B. firmus-Ientus*, *B. flexus*, *B. freudenreichii*, *B. laterosporus*, *B. licheniformis*, *B. megaterium*, *B. pasteurii* and *B. subtilis* [87]. In *B. subtilis*, the ratio *C15 iso/C15 anteiso* is typically lower than in other species of Cluster I, aiding in its identification. However, strains of *B. subtilis* showed considerable variability and were subdivided into two subgroups [87].

According to fatty acid profile analysis using the Midi Sherlock system v4.0 (Microbial ID, Newark, DE, USA), the studied BMR2 isolate strain was identified as *Bacillus subtilis* with a high level of similarity index of 0.893, with 0.417 separation to the second choice, Table 5, confirming consistency between sequencing and phenotypic tests. The similarity index expresses how closely the fatty acid composition of an unknown compares with the mean fatty acid composition of the strains used to create the library; it is an expression of the relative distance of the unknown sample from the population mean and not a probability or percentage [92]. Samples with a similarity index of 0.50 or higher and with a separation of 0.10 between the first and second choice are considered good library comparisons [92]. Gudzenko et al. [89] accepted a similarity index of 0.563 as a high level to identify a *B. subtilis* ONU551 strain that degrades phenol isolated from a wastewater pharmaceutical plant [89].

The *Bacillus subtilis* ability to naturally separate proteins from cellular components into the extracellular medium allows for better protein stability conditions compared to the reducing medium of the cytoplasm, thus simplifying the methods for isolating and purifying proteases [93,94,95]. The production of proteases by the selected best proteolytic strain was studied and confirmed by submerged fermentation (Figure 1). Moreover, Lageiro et al., 2024 and 2025 [46,47] presented protease applications using the bulk proteolytic broth produced in a bioreactor by the isolated microorganism with the best proteolytic activity, analysing the proteolytic broth application in the leather tanning process and the removal of stains from cotton fabric [46,47]. Due to the demonstrated application of bulk proteases, as claimed by Alam et al. [96], the BMR2 isolate produced proteases that are very promising to be used in the leather and detergent industries.

Despite innovative bacterial and fungal enzymes being available, pancreatic bating agents remain a strong choice in the industrial leather market, despite being less sustainable, due to their ability to deliver a well-balanced combination of diverse proteases, paired with lipase content, making them particularly effective and reliable for various tanning applications [97]. Besides the sustainability issue, the use of bacterial enzymes usage presents other advantages over the pancreatic enzymes more commonly used in the industry. They are better adapted to industrial conditions and result in better leather physical properties (as softness and flexibility). Alam et al., 2024 [19] and Lageiro et al., 2024 [46] showed in their studies that the finished leather obtained from the bacterial enzymatic process exhibited superior mechanical properties compared to the conventional pancreatic enzymatic process. The less sustainable option of using pancreatic cocktails reinforces innovation in enzyme technology.

The use of alkaline microbial proteases makes the industry eco-sustainable [2], both in terms of reducing chemical consumption and lowering effluent treatment costs, as well as improving the final product’s properties. The produced microbial proteases present significant potential for sustainability and green chemistry, with possible applications in tanneries, the detergent industry or proteinaceous effluent and waste treatments due to their proteolytic activity and adaptation to industrial conditions.

Future work should focus on optimising fermentation parameters, scaling up production, and characterising the enzymatic properties in detail to support commercial applications.

Further characterisation of the proteases produced by *Bacillus subtilis* CCMI 1253 (BMR2) is essential, particularly regarding their biochemical properties, and enzyme purification to understand the potential for industrial formulation.

Pilot-scale fermentation trials and downstream processing studies will be crucial for evaluating the scalability and cost-effectiveness of the production process. Furthermore, application-based validation of the crude and/or purified enzyme in relevant industrial processes, such as leather processing, detergent formulation, and treatment of proteinaceous waste, should be conducted to confirm functional performance under operational conditions.

## 4. Materials and Methods

All reactants and solvents used were of reagent or analytical grade (Merck, Laborspirit, Lisbon, Portugal). All solutions were prepared with purified water (Millipore Elix Essential 10, Burlington, MA, USA). All culture media (BD Difco, Detroit, MI, USA) were sterilised in an autoclave (AJC, Uniclave 88, Cacém, Portugal) for 20 min at 121 °C.

### 4.1. Screening and Isolation of Proteolytic Microorganisms

The screening of proteolytic microorganisms from alkaline industrial tanning baths at the Portuguese agroindustry (Monteiro Ribas, Porto, Portugal) was conducted to obtain strains with proteolytic activity that were already adapted to extreme industrial conditions.

Samples were collected from the tanks of soaking, purging and liming located at the beamhouse pretanning building (N41°10′59.925″, W8°37′2.939″) of the mentioned leather agroindustry. The soaking, purge, and liming alkaline bath samples were collected in sterile flasks and transported to the laboratory in liquid nitrogen and stored at 4 °C (Radiber Sa, UKS-5000, Barcelona, Spain) and −80 °C (Sanyo Electric, Ultra-low temperature freezer MDF-592, Osaka, Japan) to further studies.

In the leather making process, the beamhouse pretanning operations consist of several steps necessary to prepare the hide for the subsequent tanning process, including cleaning the hide to remove all hair and dirt.

Soaking, liming, and deliming/bating are key beamhouse operations. Soaking rehydrates hides and removes salt. Liming removes hair from the hide and opens up the hide fibre. Deliming and bating had the purpose to adjust the pH for tanning and soften the hide; both these steps generated the purge bath. Deliming reduces the alkalinity of the hide after liming, preparing it for tanning. In bating, enzymes may be used to soften the hide and remove any remaining unwanted substances. At the time of sampling, the soaking and liming baths were at 27 °C, and the purge bath from the deliming and bating steps was at 30 °C. The pH of the samples was assessed with a potentiometer (Crison pH meter Basic 20, Barcelona, Spain). For screening assays the four agro-industrial baths samples were individually diluted (10^0^, 10^−1^, 10^−2^, 10^−3^, 10^−4^, 10^−5^) and inoculated into identified polystyrene Petri dishes of 9 cm diameter (DeltaLab, Barcelona; Spain) with different commercial culture media, pH and incubation temperature: nutrient agar [41] (NA, pH 7.4), skim milk agar [41] (SMA, pH 7.0), alkaline Bacillus medium (ATCC 661, pH 7.4) and yeast malt agar [98,99] (YM agar, pH 6.2), were incubated (WTE Binder B53 incubator, Tuttlingen, Germany) at 37 °C for NA, ATCC 661 and SMA culture media and at 28 °C for YM agar grow medium. After 24 h growth time, the formed colonies were counted as colony forming units, CFU, a measure of the number of viable clonogenic cells, which indicates the number of cells that remain viable enough to proliferate and form small colonies [100].

The four isolates with the larger halos in the SMA plates were named BMR1 to BMR4 and selected for further proteolytic tests.

All four isolates were monthly inoculated and preserved in Petri dishes or slants with NA medium and stored for preservation [41] at −20 °C (Bosch, KGS3722, Stuttgart, Germany) and −80 °C in the ultra-low temperature freezer.

### 4.2. Proteolytic Activity and Growth of the Isolated Microorganisms

The selected isolates (BMR1 to BMR4) were reinoculated onto solid SMA and NA plates at 30° C, 37 °C, and 50 °C and observed for growth and proteolytic activity.

#### 4.2.1. Protease Production in Solid Medium

The evaluation of the proteolytic (caseinolytic) activity of the isolates (BMR1 to BMR4) was performed by visualising the halo formation in skim milk agar (SMA) medium during 3 days at 30, 37 and 50 °C [37,41,101]. The clearance zones around the bacterial colonies, halo indicative of proteolysis, were measured using a ruler. Bacterial isolates manifesting substantial clear zones (more than 15 mm) were chosen for further identification [37].

#### 4.2.2. Protease Production in Liquid Medium

One colony of each isolate (BMR1 to BMR4) from the SMA plates, was inoculated into liquid growth medium in a 100 mL shake flask containing 20 mL of nutrient broth (NB) and incubated in an orbital shaker (Infors HT Multitron, Bottmingen, Switzerland) at 37 °C and 180 rpm (rotations per minute) for 24 h. Samples were taken immediately after inoculation, and at 5, 7.5, 10, 12.5, 15, and 20 h to determine proteolytic activity and cell growth. Two parallel experiments were performed for each isolate.

Proteolytic activity was determined spectrophotometrically (Jasco UV/Vis 7800, Tokyo, Japan) by the Anson 1938 and Folin and Ciocalteu 1927 method [49,50]. A calibration curve with L-tyrosine in the range of 0 to 55 μmol L^−1^ was performed at 660 nm (absorvance at 660 nm = 0.0047 × L-tyrosine (μmol L^−1^) + 0.0044, with R = 0.997) using triplicate determinations. The method limit of quantification was 0.08 U mL^−1^. One unit of proteolytic activity (U) was defined as the amount of enzyme required to hydrolyse 1.0 µmol of L-tyrosine per minute at pH 7.5 and a temperature of 37 °C by the hydrolysis of casein at a wavelength of 660 nm [49,50].

Biomass growth was assessed by cell counting with a hemocytometer [102] (Thoma chamber, Brand, Wertheim, Germany) using a phase-contrast optical microscope (400× magnification, Olympus BH-2, Tokyo, Japan). The culture specific growth rate, µ (Equation (1)), and doubling time, *t*_d_ (Equation (2)), were determined using the Monod method [103,104,105], where *X_t_* is the cell count at time *t* and *X*_0_ is the initial cell count. The specific maximum growth rate (µ_max_ corresponding to the maximum slope of the linear regression between *ln X_t_*/*X*_0_ and time *t*) was calculated for BMR1 to BMR4 isolates.

*ln X_t_* = *ln X*_0_ + µ × *t* (1)

*t*_d_ = *ln* 2/µ (2)

The isolates BMR1 and BMR2 were selected for further identification due to their higher extracellular proteolytic activity and ability to grow in solid and liquid medium.

### 4.3. Microorganism Identification

The selected BMR1 and BMR2 isolates’ fresh cell preparations were examined for morphological, physiological, biochemical, and genetic characteristics identification.

#### 4.3.1. Morphological, Physiological and Biochemical Identification

The microorganisms were subjected to several characterisation tests, including traditional Gram staining tests with triphenyl tetrazolium plate staining [37,106,107,108], and malachite green staining tests to detect the formation of endospores [37,109].

The morphological, physiological, and biochemical characteristics of the isolates were tested according to Bergey’s Manual of Systematic Bacteriology [37,71].

The microscopic cell visualisation was performed using a phase-contrast optical microscope (200 to 1000× magnification).

Colour, shape, transparency and margin were examined and recorded as colony morphological characteristics according to Masi et al., 2021 and Al-Dhabaan, 2019 [37,78]. The various morphological features, including cell shape, cell size, colonial morphology, cell motility, staining behaviour, endospore formation, spore morphology and location, and colour were examined and recorded [37,78].

The isolates were also identified using the biochemical API 20E and API 50CH tests (Analytical Profile Index, API, BioMérieux, Lyon, France), as per the API kit specific instructions [51]. Briefly, a single isolated colony (from a pure culture) was picked to make a suspension in sterile distilled water. Using a Pasteur pipette, the kit compartments were filled up (up to the brim) with the bacterial suspension. After all kit procedures, they were incubated at 37 °C for 24 to 48 h. The API test results after 24 and 48 h were registered and interpreted using a database accessed through bioMérieux’s APILAB Plus v. 3.2.2 and APIWEB™ service version 4.1.

#### 4.3.2. Molecular Identification by 16S rRNA Gene Sequencing

For the molecular identification of BMR1 and BMR2 isolates, genomic DNA was extracted using an innuPrepBacteria DNA kit (Analytic Jena Biosolution K24, Germany) from a cellular pellet obtained after cell culture in a nutrient medium, according to the kit’s instructions. Genomic DNA was isolated and amplified using universal primers from DNA-Technology A/S (Aarhus, Denmark): the fourfold-degenerate forward primer 27F-YM (5′-AGAGTTTGATYMTGGCTCAG-3′) [110,111] and reverse primer 1492R-Y (5′-TACGGYTACCTTGTTACGACTT-3′) [10,112,113], were Y is C or T and M is A or C, in a PCR reaction mixture containing Taq DNA polymerase (Biorad, Hercules, CA, USA).

PCR reaction contained 5–10 pmol of each primer, 12.5 μL of Dreamtaq master mix (Thermo Fisher Scientific, Waltham, MA, USA) and 20–40 ng of template DNA in a total volume of 25 μL.

The PCR program consisted of the following steps for a total of 35 cycles: initial denaturation at 95 °C for 5 min, denaturation at 95 °C for 30 s, hybridisation at 50 °C for 30 s, and elongation at 72 °C for 45 s followed by a 10 min extension. A purification step using exoSAP was performed before sequencing.

The PCR amplified products were sequenced through commercial services provided by StabVida (Caparica, Portugal). The obtained nucleotide sequences were directly compared against the known sequences in the GenBank database (https://www.ncbi.nlm.nih.gov/genbank/, accessed on 25 March 2025) [10]. The comparison of sequencing results was performed using BLAST through NCBI’s 16S ribosomal RNA sequences database (Bacteria and Archaea).

Multiple sequence alignment was performed using the Clustal Omega Multiple Sequence Alignment (MSA, EMBL-EBI, European Bioinformatics Institute, Cambridgeshire, UK). A phylogenetic tree was constructed using the neighbour-joining method [52,114,115,116,117,118].

#### 4.3.3. Fatty Acid Methyl Esters Analysis Identification

Bacterial identification of the isolated BMR2 was also performed using the cell lipids Gas Chromatography analysis of Fatty Acid Methyl Esters (GC-FAME) at the Microbial ID, Inc. (Newark, DE, USA) using the TSBA40 method (Microbial ID, Inc., Newark, DE, USA). The GC-FAME profile of the isolated BMR2 was introduced into the Sherlock version 4.0 Microbial Identification System (MIDI Inc., Newark, DE, USA) for microorganism identification [92,119,120,121,122,123,124].

### 4.4. Statistical Analysis

Means and standard deviations were calculated using one-way ANOVA, and the Tukey HSD test was applied for *p* < 0.05 (*n* = 3) using the Statistica software, v. 8.0 (StatSoft, Tulsa, OK, USA). Graphics with standard deviation error bars were created using Excel software (Office 2019, Microsoft Corporation, Redmond, WA, USA).

## 5. Conclusions

The screening of proteolytic microorganisms from the leather industry processing baths was conducted to produce proteases adapted to these specific industrial conditions.

Two bacterial isolates were isolated from the purge alkaline bath of a Portuguese leather factory, exhibiting extracellular proteolytic bulk activities of 0.51 U mL^−1^ (BMR1) and 0.70 U mL^−1^ (BMR2) within only 7.5 h of fermentation.

Both isolates were identified as *Bacillus subtilis* CCMI1254 (BMR1) and *Bacillus subtilis* CCMI1253 (BMR2). A combination of identification methods (morphological, physiological, biochemical, and genetic analysis) was employed to confirm the identification as *Bacillus subtilis*.

*Bacillus subtilis* CCMI 1253 (BMR2) exhibited the most promising proteolytic activity and growth rate, confirming its suitability for protease production under submerged fermentation.

The identified strain demonstrated significant potential for industrial applications due to its ability to produce extracellular alkaline proteases that remain active and stable under harsh conditions. The findings reinforce the relevance of exploring extreme and underutilised environments, such as tannery alkaline baths, for the discovery of robust microbial strains with high biotechnological value.

The produced microbial proteases present potential for sustainability and green chemistry, with possible industrial applications in leather and detergent industries or proteinaceous effluent and waste treatments due to their proteolytic activity and adaptation to industrial conditions. Industrial applications of microbial proteases create a path toward sustainability and the circular economy.

## Figures and Tables

**Table 1:** Microorganism plate colonies counting of the proteolytic microorganism screening from three alkaline tannery baths: soaking, purge and liming baths, inoculated on a Petri dish with different media, pH and temperature growth, with 24 h growth at the indicated conditions and 10^−2^ bath dilution.

Medium Type (Conditions) ^(1)^	Soaking Bath pH 9.03	Purge Bath pH 9.45	Liming Bath pH 12.62
**NA (pH 7.4–37 °C) ^(2)^**	+	+	−
**ATCC 661 (pH 7.4–37 °C) ^(2)^**	−	−	+
**SMA (pH 7.0–37 °C) ^(3)^**	>300 CFU (7 CFU with halos)	11 CFU (4 CFU with big halos)	−
**YM agar (pH 6.2–28 °C) ^(2)^**	−	−	−

^(1)^ NA—Nutrient agar; SMA—Skim milk agar; YM—Yeast malt; ATCC 661—Alkaline *Bacillus* medium. ATCC—American Type Culture Collection. ^(2)^ The sign + means microorganisms growth without halos, and − means no growth. ^(3)^ >300 CFU means more than 300 CFU (Colony Forming Unit).

**Table 2:** Proteolytic halos dimensions of the purge tanning bath isolates in SMA medium at 37 °C.

Isolate	Halo Diameter (mm)
BMR1	16.9 ± 2.2 ^b^
BMR2	30.1 ± 1.4 ^a^
BMR3	9.2 ± 1.3 ^c^
BMR4	12.3 ± 1.3 ^c^

Average halo diameter in mm for three SMA plates, with three colonies per plate. Standard deviation, as determined by Analysis of Variance (ANOVA) (*p* < 0.05, *n* = 3) and the Tukey HSD (Honestly Significant Difference) test (different letter indexes indicate significant differences).

**Table 3:** Proteolytic isolates characterisation test results.

Test	BMR1 ^(1)^	BMR2 ^(1)^
Cell shape	rod	rod
Chains of cells	yes	yes
Gram staining	+	+
Colony in NA	yes	yes
Motility	yes	yes
Endospores	yes, apical	yes
Casein hydrolysis	yes	yes

^(1)^ + shows a positive result.

**Table 4:** API 20E and API 50CHB/E positive test results for the BMR1 and BMR2 proteolytic isolates.

API Test	Test *	BMR1 ^(1)^	BMR2 ^(1)^
20E	ONPG ^(2)^	+	+
	L-arginine	+	−
	Citrate (Simmons’)	+	−
	Urease	+	+
	Sodium pyruvate	+	+
	Gelatinase	+	+
	Oxidase	+	+
**API Test**	Test *	**BMR1 ^(1)^**	**BMR2 ^(1)^**
50 CHB/E	Glycerol	+	+
	L-Arabinose	+	+
	Ribose	+	+
	D-Xylose	+	+
	D-Glucose	+	+
	D-Fructose	+	+
	D-Mannose	+	+
	Inositol	+	+
	Mannitol	+	+
	Sorbitol	+	+
	α-Methyl-D-glucoside	+	+
	Amygdalin	+	+
	Arbutin	+	+
	Aesculin	+	+
	Salicin	+	+
	Cellobiose	+	+
	Maltose	+	+
	Melibiose	+	+
	Sucrose	+	+
	Trehalose	+	+
	D-Raffinose	+	+
	D-Turanose	+	+

* The tests with negative results for both isolates were not listed. *Bacillus* strains identification reference results using the API system were presented in Logan and Berkeley, 1984 [51]. ^(1)^ + shows a positive result; − shows a negative result. ^(2)^ ONPG: 2-nitrophenyl-βD-galactopyranoside.

**Table 5:** GC-FAME fatty acid reference peaks and percentages obtained from the BMR2 chromatogram.

Retention Time (min)	Reference Peak	BMR2 Isolate (%)
5.647	13:0 anteiso	0.13
6.812	14:0 iso	0.80
7.316	14:0	0.28
8.261	15:0 iso	14.06
8.398	15:0 anteiso	45.10
9.858	16:0 iso	3.24
10.470	16:0	2.47
11.548	17:0 iso	7.88
11.707	17:0 anteiso	18.75
Σ *iso*, *anteiso*		90%
*C15 iso*/*C15 anteiso*		0.31
BMR2 Similarity Index * with *Bacillus subtilis*	0.893	

* Similarity Index obtained from Sherlock version 4.0 Microbial Identification System (MIDI Inc., Newark, DE, USA). *Bacillus apiarus* as a second choice with a 0.476 similarity index, presenting 0.417 separation from the first choice (*Bacillus subtilis*).

## Data Availability

The original contributions presented in the study are included in the article, further inquiries can be directed to the corresponding author.

## Supplementary Materials

The following supporting information can be downloaded at https://www.mdpi.com/article/10.3390/molecules30173632/s1, Table S1: Identity matrix of the isolates BMR1 and BMR2.
