# Supplementary material for: Proteolytic Bacillus sp. Isolation and Identification from Tannery Alkaline Baths
Source: Molecules. 2025 Sep 5;30(17):3632. doi: 10.3390/molecules30173632 (PMC12430094; doi:10.3390/molecules30173632)
Supplement: Supplementary file 1 [file molecules-30-03632-s001.zip › molecules-3810750-supplementary.pdf]

Supplementary material to the paper entitled:

Proteolytic *Bacillus sp.* isolation and identification from tannery alkaline baths

Table S1. Identity matrix of the isolates BMR1 and BMR2, from Clustal Omega Multiple Sequence Alignment (MSA) (EMBL-EBI, European Molecular Biology Laboratory, Cambridgeshire, UK) with 21 GenBank accession numbers (retrieved from NCBI Blast, https://blast.ncbi.nlm.nih.gov, accessed on 25 March 2025. Ass. N. KP729612.1: *Bacillus cereus* FT1, a proteolytic microorganism, as the outsider) [52].

#	*Bacillus*	Ass. N.	*1*	2	3	4	5	6	7	8	9	10	11	12	13	14	15	16	17	18	19	20	21
1	*cereus*	KP729612.1	100	91.37	91.44	91.03	90.87	91.16	90.89	90.89	90.89	90.88	91.56	90.97	90.81	90.97	90.97	90.97	90.89	90.97	90.97	90.82	90.82
2	*stercoris*	NR_181952.1	91.37	100	99.3	98.9	98.55	98.96	98.76	98.49	99.03	98.42	99.72	98.55	98.27	99.04	99.04	98.97	98.9	98.69	98.69	98.69	98.69
**3**	**BMR1***	PQ326426.1	**91.44**	99.3	100	99.51	99.51	99.51	99.51	99.51	99.51	99.51	**99.86**	99.51	98.46	**99.23**	**99.23**	99.3	99.23	99.44	99.44	99.44	99.44
4	*halotolerans*	MF988689.1	91.03	98.9	99.51	100	98.9	100	99.18	99.66	99.93	98.63	100	99.17	98.82	98.9	98.9	98.97	98.9	99.11	99.11	99.11	99.11
5	*halotolerans*	PQ344167.1	90.87	98.55	99.51	98.9	100	99.03	98.69	98.97	99.37	99.31	100	99.45	98.26	98.42	98.42	98.49	98.42	98.62	98.62	98.62	98.62
6	*halotolerans*	MK517597.1	91.16	98.96	99.51	100	99.03	100	99.45	99.79	100	98.83	100	99.38	98.89	99.17	99.17	99.24	99.17	99.38	99.38	99.38	99.38
7	sp. 43A	PV264819.1	90.89	98.76	99.51	99.18	98.69	99.45	100	99.25	100	98.7	100	99.04	98.89	99.73	99.73	99.8	99.73	99.93	99.93	99.93	99.93
8	sp. 19D1S38	MN620404.1	90.89	98.49	99.51	99.66	98.97	99.79	99.25	100	100	98.56	100	98.97	98.9	98.98	98.98	99.05	98.98	99.18	99.18	99.18	99.18
9	sp. Wb-5	PP962457.1	90.89	99.03	99.51	99.93	99.37	100	100	100	100	99.31	100	99.38	98.96	99.72	99.72	99.79	99.72	99.93	99.93	99.93	99.93
10	sp HS6-2	PQ056040.1	90.88	98.42	99.51	98.63	99.31	98.83	98.7	98.56	99.31	100	100	99.31	98.2	98.42	98.42	98.49	98.42	98.63	98.63	98.63	98.63
**11**	**BMR2***	PQ325245.1	**91.56**	99.72	**99.86**	100	100	100	100	100	100	100	100	100	98.95	**99.72**	**99.72**	99.79	99.72	99.93	99.93	99.93	99.93
12	sp. 10-6	PQ056104.1	90.97	98.55	99.51	99.17	99.45	99.38	99.04	98.97	99.38	99.31	100	100	98.27	98.76	98.76	98.83	98.76	98.97	98.97	98.97	98.97
13	*tequilensis*	NR_104919.1	90.81	98.27	98.46	98.82	98.26	98.89	98.89	98.9	98.96	98.2	98.95	98.27	100	98.9	98.96	98.83	98.9	98.76	98.83	98.96	98.9
14	*subtilis* **	NR_027552.1	90.97	99.04	99.23	98.9	98.42	99.17	99.73	98.98	99.72	98.42	99.72	98.76	98.9	100	100	99.93	99.87	99.67	99.66	99.66	99.67
15	*subtilis* **	NR_113265.1	90.97	99.04	99.23	98.9	98.42	99.17	99.73	98.98	99.72	98.42	99.72	98.76	98.96	100	100	99.93	99.86	99.66	99.66	99.66	99.66
16	*inaquosorum*	NR_104873.1	90.97	98.97	99.3	98.97	98.49	99.24	99.8	99.05	99.79	98.49	99.79	98.83	98.83	99.93	99.93	100	99.93	99.74	99.73	99.73	99.74
17	*rugosus*	NR_181236.1	90.89	98.9	99.23	98.9	98.42	99.17	99.73	98.98	99.72	98.42	99.72	98.76	98.9	99.87	99.86	99.93	100	99.67	99.66	99.8	99.81
18	*mojavensis*	NR_024693.1	90.97	98.69	99.44	99.11	98.62	99.38	99.93	99.18	99.93	98.63	99.93	98.97	98.76	99.67	99.66	99.74	99.67	100	100	99.86	99.87
19	*mojavensis*	NR_112725.1	90.97	98.69	99.44	99.11	98.62	99.38	99.93	99.18	99.93	98.63	99.93	98.97	98.83	99.66	99.66	99.73	99.66	100	100	99.86	99.86
20	*halotolerans*	NR_115929.1	90.82	98.69	99.44	99.11	98.62	99.38	99.93	99.18	99.93	98.63	99.93	98.97	98.96	99.66	99.66	99.73	99.8	99.86	99.86	100	100
21	*halotolerans*	NR_115063.1	90.82	98.69	99.44	99.11	98.62	99.38	99.93	99.18	99.93	98.63	99.93	98.97	98.9	99.67	99.66	99.74	99.81	99.87	99.86	100	100

#**: 1:** KP729612.1 – *B. cereus* FT1; **2**: NR_181952.1 - *B. stercoris* D7XPN1; **3**: PQ326426.1 - *Bacillus subtilis* CCMI1254 (BMR1); 4: MF988689.1 – *B. halotolerans* 1-1; **5**: PQ344167.1 – *B. halotolerans* SM519.1; **6**: MK517597.1 – *B. halotolerans* PL-3; **7**: PV264819.1 – *B.* sp. 43A; **8**: MN620404.1 – *B.* sp.19D1S38; **9**: PP962457.1 – *B.* sp. Wb-5; **10**: PQ056040.1 - B. sp. HS6-2; **11**: PQ325245.1 - *Bacillus subtilis* CCMI1253 (BMR2); **12**: PQ056104.1 – *B.* sp. 10-6; **13**: NR_104919.1 – *B. tequilensis* 10b **14**: NR_027552.1 – *B. subtilis* JCM1465; **15**: NR_113265.1 – *B. subtilis* DSM11; **16**: NR_104873.1 - *B. inaquosorum* BGSC 3A28; **17**: NR_181236.1 – *B. rugous* SPB7; **18:** NR_024693.1 – *B. mojavensis* IFO 15718; **19:** NR_112725.1 – *B. mojavensis* NBRC; **20:** NR_115929.1 – *B. halotolerans* LMG 22476; **21:** NR_115063.1 – *B. halotolerans* DSM 8802..

*# **3**: PQ326426.1 - *Bacillus subtilis* CCMI1254 (BMR1) and #**11**: PQ325245.1 - *Bacillus subtilis* CCMI1253 (BMR2) had **99.86% identity**.

** #**3**: PQ326426.1 - *Bacillus subtilis* CCMI1254 (BMR1) had **99.23% identity** with #**14**: NR_027552.1 – *B. subtilis* JCM1465 and #**15**: NR_113265.1 – *B. subtilis* DSM11. #**11**: PQ325245.1 - *Bacillus subtilis* CCMI1253 (BMR2) had **99.72% identity** with #**14**: NR_027552.1 – *B. subtilis* JCM1465 and #**15**: NR_113265.1 – *B. subtilis* DSM11; **99.93% identity** with #**18**: NR_024693.1 – *B. mojavensis* IFO 15718; #**19**: NR_112725.1 – *B. mojavensis* NBRC; #**20**: NR_115929.1 – *B. halotolerans* LMG 22476;# **21**: NR_115063.1 – *B. halotolerans* DSM 8802; and **100% identity** with #**4**: MF988689.1 – *B. halotolerans* 1-1; #**5**: PQ344167.1 – *B. halotolerans* SM519.1; #**6**: MK517597.1 – *B. halotolerans* PL-3 and #**10**: PQ056040.1 - *B.* sp HS6-2.
